# Cognitive Emotion Regulation Strategies as Mediators between Resilience and Stress during COVID-19 Pandemic

**DOI:** 10.3390/ijerph191912631

**Published:** 2022-10-03

**Authors:** Andreea Ursu, Cornelia Măirean

**Affiliations:** 1 Faculty of Educational Sciences, Ștefan cel Mare University of Suceava, 720229 Suceava, Romania; 2 Faculty of Psychology and Educational Sciences, Alexandru Ioan Cuza University of Iași, 700554 Iași, Romania

**Keywords:** resilience, cognitive emotion regulation, stress, COVID-19 pandemic, young adults

## Abstract

(1) Background: Although there is accumulating evidence for the associations between resilience, emotion regulation and stress, little is known about the mechanisms of these relations. To extend the existing research, the present study examined cognitive emotion regulation strategies as one potential mechanism between trait resilience and perceived stress during the COVID-19 pandemic. (2) Methods: Young adults (N = 266; M = 20.05; SD = 3.93) were invited to fill out questionnaires that assessed trait resilience, cognitive emotion regulation strategies and perceived stress. (3) Results: The results showed that resilience was negatively associated with perceived stress and with self-blame, catastrophizing and rumination, and positively associated with positive reappraisal, focus on planning, positive refocus and putting into perspective. Stress was positively associated with self-blame, catastrophizing, rumination, other-blame and acceptance, and negatively associated with positive reappraisal and positive refocus. Moreover, positive refocus, rumination, catastrophizing and self-blame partially explained the associations between trait resilience and perceived stress during the COVID-19 pandemic. (4) Conclusions: These findings highlight the potential utility of targeting cognitive emotion regulation strategies in the development and implementation of preventive interventions for reducing stress during highly challenging situations.

## 1. Introduction

The World Health Organization (WHO) recognized the Coronavirus Disease 2019 (COVID-19) as a pandemic on 11 March 2020 [1]. Many countries, including Romania, have taken stringent steps to impede the spread of infection throughout the population. In Romania, the nationwide lockdown was implemented on 16 March 2020, when the country was already in its third infection scenario with 168 confirmed COVID-19 cases [2]. Taking into account the high level of psychological problem prevalence, it is crucial to understand the factors that contribute to people’s stress during the COVID-19 pandemic in order to avoid such mental health difficulties in the future. In our study, we focused on the relation between resilience and stress, taking into account the role of emotion regulation in explaining the connection between resilience and perceived stress.

### 1.1. Stress Generated by the COVID-19 Pandemic

Stress was defined as the physical and/or psychological response to environmental stressors, defined as external demand on an individual [3]. According to Thoits [4], the COVID-19 pandemic represents a traumatic stressor, as it is threatening people’s lives, bodily integrity and sanity. Stress has been associated with worse health outcomes as well as with low levels of quality of life and low daily functioning. Existing research broadly supports the positive link between stress and psychological outcomes. Higher stress is related to high depression and low life satisfaction among students [5], high level of anxiety among adults [6], low quality of life, high levels of insomnia and burnout among university students [7] and increased risk of cardiovascular diseases [8,9].

COVID-19, with its high rate of infection, mortality and its related measures (lockdown, quarantines, self-isolation), caused a variety of mental health problems and stress [10,11]. According to the results of one meta-analysis (66 studies; 221.970 participants), during the COVID-19 pandemic, the prevalence of distress was 41.1% [12].

### 1.2. Resilience and Stress during the COVID-19 Pandemic

Researchers conceptualized resilience as a trait, as an outcome and as a process. Resilience as a trait supposes that one experiences mild, momentary distress following adversity [13]; resilience as an outcome supposes that one flourishes following adverse events [14]; and resilience as a process involves positive adaptation after trauma [15]. In this paper, we define resilience as a personal factor that enables individuals to cope with adversity (trauma, illness, stress) and to achieve positive growth and development [16,17].

Defined as a psychological resource, resilience enables a person to successfully adapt to major stressors [18]. According to this approach, resilience may contribute to optimal mental health during times of stress and has a positive effect on a variety of mental health and well-being outcomes [19]. More precisely, trait resilience was positively associated with life satisfaction [20], positive affect, flourishing [21] and psychological wellbeing [22] and negatively associated with anxiety, depression and social dysfunction [22,23,24]. In addition, the results of a meta-analysis (60 studies) showed that trait resilience is negatively correlated with depression, anxiety and negative affect and positively correlated with life satisfaction and positive affect [25]. In a study on Romanian adults, Stănculescu [26] found that trait resilience was negatively correlated with fear of COVID-19. In addition, Cazan and Truta [27] found that trait resilience was positively correlated with life satisfaction, and negatively correlated with conflicts and frustrations as dimensions of student life stressors.

Individuals with a low level of resilience are more vulnerable to experiencing stress [28]. As a result, resilience can be viewed as a protective factor in times of stress and one that decreases the prevalence of psychological problems [22,23,24]. The negative relationships between resilience and stress during the COVID-19 pandemic have been reported in previous studies on university students [29,30]. However, the mechanisms linking resilience and stress were less investigated.

### 1.3. The Role of Emotion Regulation

Emotion regulation consists of “all the extrinsic and intrinsic processes responsible for monitoring, evaluating, and modifying emotional reactions, especially their intensive and temporal features” [31,32] (p. 27). Cognitive emotion regulation can be considered part of this broader concept and refers to conscious and cognitive ways of dealing with the intake of emotionally arousing information [33,34].

The Cognitive Emotion Regulation Questionnaire was developed [33] in order to evaluate nine cognitive emotion regulation strategies, each referring to what one person thinks during and after experiencing stressful or threatening events. Self-blame refers to blaming yourself for what you have gone through, while other-blame refers to the idea of blaming the environment or another person for something you have gone through. Rumination, also known as focus on thought, is the act of reflecting on the feelings and thoughts associated with the negative event. Catastrophizing refers to thoughts that emphasize the horror of what you have gone through. Putting into perspective is a coping strategy that involves the idea of brushing away the importance of the event/emphasizing its relativity by comparing the event to other events. Positive refocusing is when you think about happy and pleasant things instead of the actual event, while positive reappraisal refers to the idea of giving a positive significance in terms of personal development to the negative event. Acceptance is defined as thinking about accepting what you have gone through and resigning yourself to what has happen. Refocusing on planning refers to thinking about what steps to do and how to handle the unfavorable event. These nine strategies are highly intercorrelated and can be grouped into two classes—adaptive strategies (acceptance, positive refocus, refocus on planning, positive reappraisal and putting into perspective) and maladaptive strategies (self-blame, rumination, catastrophizing and blaming others) [33,35].

Previous studies showed the link between individual characteristics and emotion regulation. For instance, the positive relationships between resilience and adaptive cognitive emotion regulation strategies (acceptance, positive refocus, refocus on planning, positive reappraisal and putting into perspective) have been reported in previous studies [36,37,38,39]. On the other hand, the negative link between resilience and non-adaptive cognitive emotion regulation strategies (self-blame, rumination, catastrophizing and blaming others) have also been reported in previous studies [36,37].

In terms of the protective effect of adaptive cognitive emotion regulation strategies on well-being and ill-being, longitudinal and cross-sectional studies suggest a negative relationship between positive reappraisal, depression and anxiety [40,41]. Studies assessing the predictive role of other adaptive cognitive emotion regulation strategies found a negative relationship between putting into perspective and depression [42]. In addition, recent studies showed that positive refocus and positive reappraisal were negatively related with anxiety/insomnia, social dysfunction and depression, refocus on planning was negatively related to social dysfunction and depression, and acceptance was negatively related to depressive symptoms [36,43]. Longitudinal studies focusing on the link between cognitive maladaptive emotion regulation strategies and ill-being suggest positive relationships between self-blame, rumination and catastrophizing and symptoms of psychopathology such as depression and anxiety [40].

With regard to the link between emotion regulation strategies and perceived stress, evidence from cross-sectional studies showed that cognitive maladaptive strategies such as other-blame positively predicted stress in a sample of university students [41]. Concerning adaptive strategies, the results of several studies showed that strategies such as positive reappraisal and positive refocus are negatively associated with perceived stress in both an university student sample and in a sample of middle-aged adults from the general population [40,41].

In another study assessing the relationships between cognitive emotion regulation strategies and experienced stress because of the COVID-19 pandemic, a positive relationship between the use of self-blame, other-blame, rumination and catastrophizing and the perceived stress was found [43,44]. Thus, as a conclusion, the previous studies suggest that by using adaptive cognitive emotion regulation strategies (positive reappraisal, positive refocus, putting into perspective, refocus on planning and acceptance) people may more easily tolerate and deal with negative life events and have better psychological functioning. In contrast, using non-adaptive cognitive emotion regulation (rumination, catastrophizing, self-blame and other-blame) facilitates the development of psychopathology.

In previous studies conducted during the current pandemic, emotion regulation was used as a mediating variable in the relation between emotional states and mental health indicators [43,45]. One previous study explored the mediating role of cognitive emotion regulation in the relation between loneliness, intolerance of uncertainty, fear of contamination and mental well-being among elderly people in the context of the COVID-19 pandemic. They found that catastrophizing mediated the relationship between feelings of loneliness and well-being, and positive refocusing mediated the relationship between perceived social support from friends, anxiety and depression [45]. More specifically, people with high levels of loneliness who were more prone to catastrophize an event reported higher levels of anxiety-related symptoms. In contrast, those with higher levels of perceived social support from friends were more likely to think about happy and pleasant things instead of the COVID-19 pandemic. Further, they presumably felt the benefits of having someone to be there for them which made them more likely to focus on the positive sides of the event resulting in less incidences of depression and anxiety. Another study found that rumination and catastrophizing exerted a mediating effect on the development of anxiety/insomnia, somatic symptoms and depression in people who had COVID-19-positive cases around them [43].

Previous research on the link between resilience, cognitive emotion regulation and perceived stress during the COVID-19 pandemic has tended to focus on (a) establishing latent models of the COVID-19 fear and its associations with mental health and potentially protective factors among university students [30] and (b) assessing the mediating role of coping strategies in the relation between COVID-19-related factors and mental health among young adults [29] and adults [43]. Moreover, previous studies investigated the mediating role of emotion regulation on the link between individual characteristics and mental health among elderly people [45], as well as the mediating role of preventive behaviors on the link between hope and resilience among adults [22]. However, previous studies did not assess the mediating role of cognitive emotion regulation in the relation between resilience and perceived stress among adults. Given the fact that cognitive emotion regulation can significantly protect psychological health [36,43,44], different cognitive emotion regulation strategies can be used to explain the relationship between trait resilience and perceived stress during the COVID-19 pandemic.

### 1.4. The Romanian COVID-19 Pandemic Context

The first COVID-19 case was confirmed on 26 February 2020, in the county of Gorj in southwestern part of Romania [46]. During 2020, two waves of COVID-19 were in the country. On 16 March 2020, during the first COVID-19 wave, a state of emergency was declared in Romania for 30 days [47]. Later on, it was extended up to 14 May 2020. The state of emergency confined people over the age of 65 to their homes; schools and universities were closed, all didactic and research activities were moved online, non-essential movement was forbidden, and non-essential businesses were closed [48]. All religious, artistic, cultural and private (weddings, baptisms, funerals) gatherings were suspended. Between 15 May 2020 and 9 March 2022 a state of alert was declared by the Romanian government. This introduced a relaxation of measures. Non-essential movement was limited, non-essential businesses were open at different percentages of capacity depending on the local situation, wearing a mask in public indoor and outdoor spaces was mandatory, and keeping a 1.5 m distance from other people was mandatory [49]. On 27 December 2020, the vaccination program started in Romania. In the first step, health workers, staff in residential and health-social centers, people at high risk of severe SARS-CoV-2 infection and staff in other key areas essential to the proper functioning of society could get the first shot. In 2021, two other waves of the pandemic hit Romania. The data collection of the present study was run during the fourth wave of the COVID-19 pandemic in Romania (July to mid-December 2021). Although during this wave the highest rate of infection and deaths was registered and a lower level of vaccinations was observed, the restrictions were eased on several occasions. In an analysis of the Romanian government’s implemented measures for lowering the pandemic effects for the fourth wave of the COVID-19 pandemic, Turi and colleagues [50] reported a stringency index between 50 and 60, which suggests a low level of restrictions related to closing schools, closing jobs, postponing community events, restricting gatherings, public transport closure, stay-at-home measures, communication campaign, international travel restrictions and international travel controls. In October, new restrictions were imposed specially for unvaccinated individuals. Stay-at home restrictions during nighttime (between 22:00 and 05:00) were imposed for unvaccinated persons, short opening hours for restaurants were imposed, and only vaccinated people could go to restaurants. Private events were forbidden. During the data collection, around 40% of Romanian people were fully vaccinated, compared to the EU average of 69%. Related to elders, only 45% of Romanians were vaccinated compared to the EU average of 89%.

### 1.5. The Present Study

The current study aims to test whether emotion regulation mediates the relation between trait resilience and perceived stress during the COVID-19 pandemic. In order to test this mediation, we hypothesize that (a) resilience will be negatively associated with perceived stress and maladaptive cognitive emotion regulation strategies (self-blame, other-blame, catastrophizing, rumination) and positively associated with adaptive cognitive emotion regulation strategies (positive reappraisal, putting into perspective, positive refocus, acceptance and refocus on planning); (b) maladaptive cognitive emotion regulation strategies (self-blame, other-blame, catastrophizing, rumination) will be positively associated with stress, while adaptive cognitive emotion regulation strategies (positive reappraisal, putting into perspective, positive refocus, acceptance and refocus on planning) will be negatively associated with stress; and (c) cognitive emotion regulation strategies will mediate the relationship between trait resilience and perceived stress.

## 2. Materials and Methods

### 2.1. Participants

The sample of the present study consists of 266 college students (83.8% women, 15.8% men and 0.4% non-binary). The age ranges between 18 and 53 years, with a mean of 20.05 (SD = 3.93). At the time of the data collection, almost eighty seven percent (*n* = 231) of the sample had completed high school level, while the other participants had completed undergraduate and graduate levels at another faculty. The majority of participants were not involved in a committed romantic relationship (64.7% being single, while 0.4% were divorced), while thirty-three percent were involved in committed non-marital relationships and three percent were married. Fifty-eight percent of the sample lived in the urban area, while the rest of participants lived in the rural area.

### 2.2. Procedure

An online survey including informed consent, socio-demographic information and the main questionnaire was created. The entire survey took approximately 10–15 min to complete. First year Psychology students from a large university in the northeastern part of Romania were invited to participate in this study. All participants signed a consent form to participate in the study. All the study questionnaires were filled in online at the beginning of the class hours. Students were told that if they did not want to participate in this study, they would receive another task for the extra course credit. No information related to their name or e-mail address was collected. The data collection was conducted between 23 November and 5 December 2021 when the average of new COVID-19 cases in Romania was around two dozen and the average reported number of deaths caused by the COVID-19 disease was around one hundred and fifty. The situation for participants was as follows: students were attending the classes online, and they could vaccinate themselves, as in October, the vaccination program was available for the general population. The majority of them were living in their hometowns with their families.

The study protocol was approved by the Ethics Committee of the university where the study was conducted.

Prior to filling out the socio-demographic and main sections of the questionnaire, participants were told that the goal of the study was to investigate the experienced emotions during the COVID-19 pandemic and how they managed those emotions. In addition, participants were told that their participation was voluntary and that their responses would be confidential and anonymous. All participants were provided with an e-mail address if they wanted to ask for more details about the study. All students received extra course credit.

### 2.3. Materials

**Socio-demographics questions.** This section included questions about participants’ gender, age, education, relationship status and area of living.

**Resilience.** A Romanian translation of the Brief Resilience Scale (BRS) [51] was used to measure resilience during the COVID-19 pandemic. First, two psychologists enrolled at doctoral programs, fluent in both English and Romanian languages, translated the BRS scale from English into Romanian. Then, a psychologist with a doctorate in psychology translated the Romanian version back into English. Finally, in order to get the final Romanian translation, the original form of the BRS was compared with this back translation version by two psychology teachers. The necessary modifications were made. BRS is a self-reported scale which consists of 6 items, assessed on a 5-point Likert scale, from 1 (strongly disagree) to 5 (strongly agree). Three out of six items are positively worded (e.g., I tend to bounce back quickly after hard times) and the other three items are negatively worded (e.g., I have a hard time making it through stressful events). The Cronbach’s alpha coefficient of BRS in this study was 0.69.

**Emotion regulation.** In order to assess the cognitive emotion regulation strategies used by participants during the COVID-19 pandemic, the Cognitive Emotion Regulation Questionnaire (CERQ) [35] adapted for the Romanian population [52] was used. Participants were asked to report how they regulated their emotions when confronted with stress caused by the COVID-19 pandemic. CERQ is a self-reported scale which consists of 36 items, grouped into nine dimensions: self-blame (e.g., I feel that I am the one who is responsible for what has happened), other-blame (e.g., I feel that others are responsible for what has happened), acceptance (e.g., I think that I have to accept that this has happened), refocus on planning (e.g., I think about a plan of what I can do best), positive refocusing (e.g., I think of pleasant things that have nothing to do with it), rumination (e.g., I often think about how I feel about what I have experienced), positive reappraisal (e.g., I think I can learn something from the situation), putting into perspective (e.g., I tell myself that there are worse things in life) and catastrophizing (e.g., I continually think how horrible the situation has been). Each item refers to what someone thinks after experiencing threatening or stressful events. Items were assessed on a 5-point Likert scale, from 1 (almost never) to 5 (almost always). In the present study, Cronbach’s Alpha values ranged between 0.68 for acceptance and 0.82 for other-blame and positive reappraisal (0.77 for self-blame, 0.82 for other-blame, 0.77 for rumination, 0.67 for catastrophizing, 0.78 for putting into perspective, 0.79 for positive refocusing, 0.82 for positive reappraisal, 0.68 for acceptance and 0.72 on refocus on planning).

**Stress.** To assess the perceived stress during the COVID-19 pandemic, the Perceived Stress Scale (PSS) [53] was used. We used the same procedure to culturally adapt the PSS scale as described in the section of the Resilience scale. We adapted the timeframe from “in the last month” to “during the COVID-19 pandemic”. Four out of ten items are positively worded (e.g., During the COVID-19 pandemic, how often have you felt confident about your ability to handle your personal problems?) while the other items are negatively worded (e.g., During the COVID-19 pandemic, how often have you felt that you were unable to control the important things in your life?). PSS is also assessed on a 5-point Likert scale between 0 (never) and 4 (very often). In this study, the Cronbach’s Alpha coefficient of PSS was 0.77.

## 3. Results

### 3.1. Data Analytic Approach

First, we conducted some preliminary analysis and the correlations between the study variables. Then, the mediational analyses were conducted using the macro PROCESS 3.5 in SPSS [54]. To determine the indirect effect of the predictor, we used 5000 bootstrap samples, and biases were corrected at 95% confidence intervals (CI). If the indirect effect confidence interval (CI) did not include zero, the indirect effect was significant at *p* < 0.05 [55]. To establish the minimal sample size necessary to test the study hypotheses, an a priori power analysis was carried out using GPower version 3.1.9.4 [56]. The results showed that a total of 100 participants was the minimum sample size needed to achieve 80% power for detecting a medium effect at a significance level of =0.05. Thus, the number of participants (*n* = 266) in this study is appropriate to test the study hypotheses.

### 3.2. Preliminary Analyses

Table 1 depicts the means, standard deviation and the minimum and maximum scores for the study’s variables. In general, the means were moderate for trait resilience, perceived stress and for several cognitive emotion regulations strategies (acceptance, rumination, positive refocus, refocus on planning, positive reappraisal and putting into perspective), while for other cognitive emotion regulation strategies (self-blame, catastrophizing and other-blame) average means were rather low. The results of an independent sample *t* test showed that there are no significant gender differences in any of the main study’s variables, all *p* > 0.05.

### 3.3. Associations between the Main Study Variables

In order to test the associations between the study variables, Pearson correlation was used. The results depicted in Table 2 suggested that the first and second hypotheses are partially supported. Trait resilience was negatively associated with perceived stress and with three out of four cognitive maladaptive emotion regulation strategies (self-blame, catastrophizing and rumination) and positively associated with four out of five cognitive adaptive emotion regulation strategies (positive reappraisal, focus on planning, positive refocus and putting into perspective). Stress was positively associated with all cognitive maladaptive emotion regulation strategies (self-blame, other-blame, catastrophizing and rumination) and one cognitive adaptive emotion regulation strategy (acceptance). Moreover, stress was negatively associated with two out of five cognitive adaptive emotion regulation strategies (positive reappraisal and positive refocus). All these results are small to medium [57].

### 3.4. Testing the Mediations

After examining the preliminary analysis, we investigated whether cognitive maladaptive and adaptive emotion regulation strategies mediated the relation between trait resilience and perceived stress. We included in the mediational analysis only the cognitive emotion regulation strategies which previously significantly correlated with both trait resilience and perceived stress. More precisely, we tested whether self-blame, rumination, positive refocus, positive reappraisal and catastrophizing mediate the relationship between trait resilience and perceived stress. In order to test these mediational analyses, the macro PROCESS in SPSS [54], model 4 (simple mediation with one mediator) was used.

#### 3.4.1. Direct Effects

Trait resilience had a direct relation with self-blame (b = −0.02, *p* = 0.0002), rumination (b = −0.22, *p* < 0.002), positive refocus (b = 0.29, *p* = 0.0003), positive reappraisal (b = 0.33, *p* < 0.001) and catastrophizing (b = −0.30, *p* < 0.001). In addition, self-blame (b = 0.10, *p* = 0.012), rumination (b = 0.22, *p* < 0.001), positive refocus (b = 0.09, *p* = 0.01), and catastrophizing (b = 0.22, *p* < 0.001) had a direct relation with perceived stress, while positive reappraisal (b = −0.02, *p* > 0.05) failed to have a direct relation with perceived stress.

#### 3.4.2. Indirect Effects

**Figure 1:**
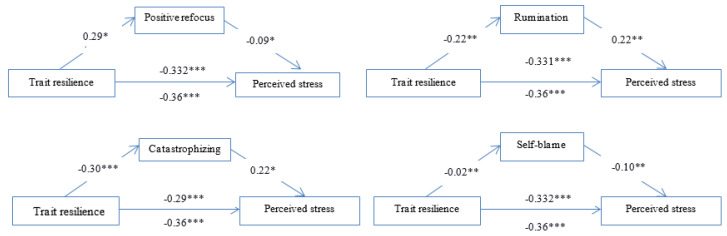
The mediation model testing cognitive emotion regulation as a mediating variable in the relationship between trait resilience and perceived stress. Note: * *p* < 0.05, ** *p* < 0.01, *** *p* < 0.001.

We assumed that cognitive emotion regulation (self-blame, rumination, positive refocus, positive reappraisal and catastrophizing) would explain the relation between trait resilience and perceived stress during the COVID-19 pandemic (Figure 1). The findings showed that positive refocus (b = −0.03; CI: −0.069; −0.003) (Figure 1a), rumination (b = −0.03; CI: −0.072; −0.006) (Figure 1b), catastrophizing (b = −0.06; CI: −0.115; −0.301) (Figure 1c) and self-blame (b = −0.03; CI: −0.067; −0.005) (Figure 1d) partially mediated the effects of trait resilience on perceived stress, while positive reappraisal (b = −0.009; CI: −0.039; 0.019) failed to mediate the effect of trait resilience on perceived stress.

## 4. Discussion

Stress can have deleterious effects on individuals. This is reflected in a rise in physical and mental health difficulties. However, previous research has shown a number of risks and protective factors. Thus, the aim of this paper was to examine the associations between trait resilience, cognitive emotion regulation strategies and perceived stress during the COVID-19 pandemic.

We first hypothesized that trait resilience would be negatively associated with perceived stress and maladaptive cognitive emotion regulation strategies (self-blame, other-blame, catastrophizing, rumination) and positively associated with adaptive cognitive emotion regulation strategies (positive reappraisal, putting into perspective, positive refocus, acceptance and refocus on planning). The results showed that trait resilience was negatively associated with perceived stress and with three out of four cognitive maladaptive emotion regulation strategies (self-blame, catastrophizing and rumination) and positively associated with four out of five cognitive adaptive emotion regulation strategies (positive reappraisal, refocus on planning, positive refocus and putting into perspective). This is in agreement with previous results [29,30] sustaining that people high on resilience reported less stress. Moreover, the results from previous studies [36,37,38,39] suggest that resilience was positively related to positive reappraisal, putting into perspective, positive refocus and refocus on planning. In addition, previous studies also reported negative associations between trait resilience and self-blame, catastrophizing and rumination [36,38]. Next, the findings of the study provided evidence supporting the following hypothesis of the study and indicated that stress was positively associated with all cognitive maladaptive emotion regulation strategies (self-blame, other-blame, catastrophizing, rumination) and with one cognitive adaptive emotion regulation (acceptance). Perceived stress was also found to be negatively associated with two out of five cognitive adaptive emotion regulation strategies (positive reappraisal and positive refocus). Similar results have also been reported in previous studies. More precisely, higher stress was related to higher use of self-blame, other-blame, catastrophizing and rumination [43,44]. Other studies suggested that when people are using positive reappraisal and positive refocus, they reported less stress [40,41].

However, some unexpected results emerged. We found a positive correlation between perceived stress and one cognitive adaptive emotion regulation (acceptance), and we did not find any significant correlation between perceived stress and two cognitive adaptive emotion regulation strategies (putting into perspective and refocus on planning). Another study, with an adult sample from India, assessing the stress caused by the COVID-19 pandemic also reported a positive correlation between acceptance and perceived stress [44]. Thus, people who tend to accept stressful situations also perceive high stress generated by these situations. It is possible that the more one attempts to accept the current situation—which includes the virus’s widespread distribution around the world, the fear of being infected by the virus, the fear of serious health problems for themselves or their close ones, the lockdown and the unavoidable shift in their basic way of life—the more powerless, stressed, and depressed they become.

Most importantly, self-blame, catastrophizing, rumination and positive refocus mediated the relationship between trait resilience and perceived stress, suggesting that the underlying mechanism between trait resilience and perceived stress can be represented by specific cognitive emotion regulation. Thus, a high level of resilience predicts a low level of engagement in non-adaptive coping strategies, such as self-blame, catastrophizing and rumination. Furthermore, these coping strategies predict a high level of stress. Moreover, resilience predicts a high level of positive refocus, which further decreases the level of perceived stress. These findings suggest that adults with high trait resilience are more likely to use cognitive adaptive emotion regulation strategies and have better psychological health. However, contrary to our expectations, positive reappraisal did not mediate the link between trait resilience and perceived stress. In the mediation analysis, this emotion regulation strategy did not predict stress. Cognitive reappraisal is an antecedent-focused strategy, typically used in the early stage of the emotion-generated process, before the occurrence of an emotional response to a particular situation [31]. During the COVID-19 pandemic, different concerns shared by the entire population all over the world probably impede the implementation of this emotion regulation strategy before the emotional response of perceived intense stress occurs. Moreover, the prolonged exposure to pandemic risks generated stress for most of the individuals, regardless of their stable tendency to use cognitive reappraisal to deal with different difficult situations. Given the fact that the causes of the emotional response of stress were too powerful, the benefits of cognitive reappraisal in early stages of generating these responses can be inhibited.

The results of the present study have several practical implications. More precisely, the results emphasize the rationale of the development and implementation of preventive interventions where people are taught to use cognitive adaptive emotion regulation strategies that were found to be beneficial for individuals’ mental health. These interventions would act as health-prevention measures, assisting people in developing a diverse set of healthy ways to deal with the diverse range of negative effects of adverse global situations such as the COVID-19 pandemic. Moreover, strategies designed to increase resilience should also focus on including psycho-education regarding the role of different cognitive emotion regulation strategies in dealing with life challenges. Currently, the majority of confinement measures are not imposed anymore. However, it is critical to design online emotion regulation training programs that have the least amount of contact with people (online format). The online format of the preventive interventions may make people feel safe and could contribute to subjective well-being and psychological health.

These findings contribute to the literature on the protective personal factors against stress in several ways. First, the present study brought additional knowledge in the area of well-being, ill-being and protective factors by showing the associations between trait resilience, adaptive and maladaptive cognitive emotion regulation strategies and perceived stress. Second, we tested the mediation role of specific cognitive emotion regulation strategies in the relation between trait resilience and perceived stress. Third, we focused on one eastern European culture, Romania, where the first case of COVID-19 was reported on 28 February 2020, while the national lockdown was instituted on the 16 March 2020.

In addition to its strengths, the present study also has some limitations. One of the most important flaws is the cross-sectional design, which does not allow us to know how the effect of these variables changes over time and does not allow us to make causal assumptions [58]. In order to understand if the mediation effect is stable over time, longitudinal designs should be used in future studies. Another limitation of this study is the use of convenience sampling for data gathering. As a result, we are unable to extrapolate the findings of this study to the broader population. However, for the group age we were interested in, the student sample is one of the most representative. Finally, we did not measure specific COVID-19-related factors (e.g., fear of COVID-19, risk perception, COVID-19 anxiety); therefore, our results could not explain how the pandemic is related to stress and resilience. Despite this limitation, our results are informative for the relation between resilience, coping strategies and stress during a highly challenging period in a vulnerable sample consisting primarily of young adults.

## 5. Conclusions

The present results highlighted, on the one hand, the positive relations between stress, self-blame, other-blame, catastrophizing, rumination and acceptance. On the other hand, stress was negatively related with trait resilience, positive reappraisal and positive refocus. Moreover, self-blame, catastrophizing, rumination and positive refocus mediated the relationship between trait resilience and perceived stress during the COVID-19 pandemic.

## Figures and Tables

**Table 1:** Descriptive statistics for study’s variables.

	M	*SD*	Min	Max
1. trait resilience	3.12	0.68	1.50	5
2. self-blame	1.99	0.81	1	4.75
3. acceptance	3.25	0.80	1	5
4. rumination	3.22	0.84	1	5
5. positive refocus	3.16	0.92	1	5
6. refocus on planning	3.47	0.78	1	5
7. positive reappraisal	3.53	0.94	1	5
8. putting in perspective	3.42	0.91	1	5
9. catastrophizing	2.19	0.79	1	4.5
10. other-blame	2.19	0.92	1	5
11. stress	3.13	0.48	1.67	5

**Table 2:** Correlations between trait resilience, cognitive emotion regulation strategies and perceived stress during the COVID-19 pandemic.

	1	2	3	4	5	6	7	8	9	10	11
1. trait resilience											
2. self-blame	−0.22 **										
3. acceptance	0.01	0.18 **									
4. rumination	−0.18 *	0.34 **	0.51 **								
5. positive refocus	0.21 **	−0.07	0.23 **	0.23 **							
6. refocus on planning	0.16 **	0.10	0.48 **	0.48 **	0.57 **						
7. positive reappraisal	0.24 **	−0.02	0.40 **	0.41 **	0.58 **	0.74 **					
8. putting in perspective	0.14 *	0.00	0.43 **	0.30 **	0.45 **	0.55 **	0.68 **				
9. catastrophizing	−0.26 **	0.39 **	0.24 **	0.32 **	−0.03	0.03	−0.05	0.00			
10. other-blame	−0.07	0.22 **	0.25 **	0.19 **	0.09	0.13 *	0.04	0.07	0.49 **		
11. stress	−0.41 **	0.22 **	0.12 *	0.24 **	−0.22 **	−0.07	−0.13 *	−0.02	0.38 **	0.24 **	

Note: *p < 0.05, ** *p* < 0.01.

## Data Availability

Data supporting the reported results can be obtained by email from the corresponding author.
